# Use of Contrast-Enhanced Ultrasonography and Shear-Wave Elastography in the Diagnosis of Granulomatous Colitis in a French Bulldog

**DOI:** 10.3390/vetsci8070133

**Published:** 2021-07-15

**Authors:** Alessia Cordella, Emmelie Stock, Isabel Van de Maele, Annelies Willems, Jimmy Saunders

**Affiliations:** 1 Department of Medical Imaging of Domestic Animals and Orthopaedics of Small Animals, Faculty of Veterinary Medicine, Ghent University, 9280 Merelbeke, Belgium; emmelie.stock@ugent.be (E.S.); jimmy.saunders@ugent.be (J.S.); 2 Small Animal Department, Faculty of Veterinary Medicine, Ghent University, 9280 Merelbeke, Belgium; i.vandemaele@ugent.be (I.V.d.M.); annelies.willems2@gmail.com (A.W.)

**Keywords:** ultrasound, CEUS, dog, colon, histiocytic ulcerative colitis

## Abstract

An 11-month-old male intact French Bulldog was referred for chronic intermittent diarrhea lasting three months. Ultrasonographic (US) examination revealed severe thickening of the wall of the colon and caecum; contrast-enhanced US (CEUS) and elastography showed increased, heterogeneous vascularization and non-uniform stiffness of the colonic wall. The mucosa was thickened, fragile, and ulcerated as revealed by endoscopy, and histological examination confirmed the suspicion of granulomatous colitis.

## 1. Introduction

Granulomatous colitis is an inflammatory condition formerly known as histiocytic ulcerative colitis or ulcerative colitis [1,2]. It is characterized by granulomatous inflammation of the mucosa, lamina propria, and submucosa of the large intestine, with the presence of a mixture of macrophages, lymphocytes, and neutrophils [3,4]. It has been described in young Boxers [1,4,5] and occasionally in other breeds, including French Bulldogs [2,6,7].

Typical clinical signs are frequent small-volume diarrhea, hematochezia, mucoid feces, and tenesmus, and the main differential diagnoses include idiopathic inflammatory bowel disease (IBD), enteric parasites or infectious agents, and neoplasia [1]. Histology of granulomatous colitis is characterized by the presence of granulomatous inflammation, particularly evident with periodic acid–Shiff (PAS) staining, in which PAS-positive material is seen in the cytoplasm of macrophages the lamina propria of the colonic wall [1,2]. In addition, in both Boxers and French Bulldogs, granulomatous colitis has been described to be associated with intramucosal E. coli, and therefore, clinical response to fluoroquinolone antimicrobials has been reported [2,8].

The clinical and histological features of granulomatous colitis have been described in detail in veterinary literature; on the contrary, detailed imaging features regarding the appearance of this disease are currently lacking.

The colonic wall can be visualized with ultrasonographic (US) examination in dogs, and in the case of granulomatous colitis, it is expected to be thickened with an irregular mucosal surface due to the presence of possible superficial erosions [9]. The US features of inflammatory and neoplastic conditions of the gastrointestinal tract often overlap, both in veterinary and human medicine. Furthermore, although widely used, the utility of abdominal ultrasonography in the clinical workup in dogs with chronic diarrhea is considered low [10,11]. In human medicine, advanced US methods, such as contrast-enhanced ultrasonography (CEUS) and elastography, are used to better characterize the different pathologies that can affect the large intestine [12,13,14].

The aim of this case report is therefore to describe the US features of a case of granulomatous colitis in a young French Bulldog, with particular emphasis on the CEUS and elastography findings.

## 2. Materials, Methods, and Results

### 2.1. Case Description and Clinical Investigations

An 11-month-old male intact French Bulldog was referred to the Veterinary Teaching Hospital of Ghent University for investigation of a 3-month history of intermittent chronic large bowel diarrhea with hematochezia and tenesmus. A hypoallergenic diet (Royal Canin Anallergenic) was fed without improvement. The dog was regularly dewormed. Fecal analysis (performed by the referring institution) showed no parasitic infestation, and the culture was negative for the presence of Salmonella, Yersinia, and Campylobacter. Physical examination revealed the presence of a mildly tense abdomen, and a hard, tubular structure was detected in the caudal abdomen on palpation. The body temperature was 38.8 °C, the heart rate was 160 bpm, and the respiratory rate was 28 rpm. The body condition score was 4/9. The rectal examination was painful and revealed severe thickening of the mucosa.

The complete blood count revealed mild monocyte leukocytosis (monocytes (/μL): 1.24, reference interval: 0.16–1.12 μL) and mild neutropenia (neutrophils (/μL): 2.14, reference interval: 2.95–11.64). The results of the biochemistry and urinalysis were unremarkable. The fecal analysis was not repeated, as it was recently performed in an external institution.

### 2.2. Imaging Diagnosis

**Figure 1:**
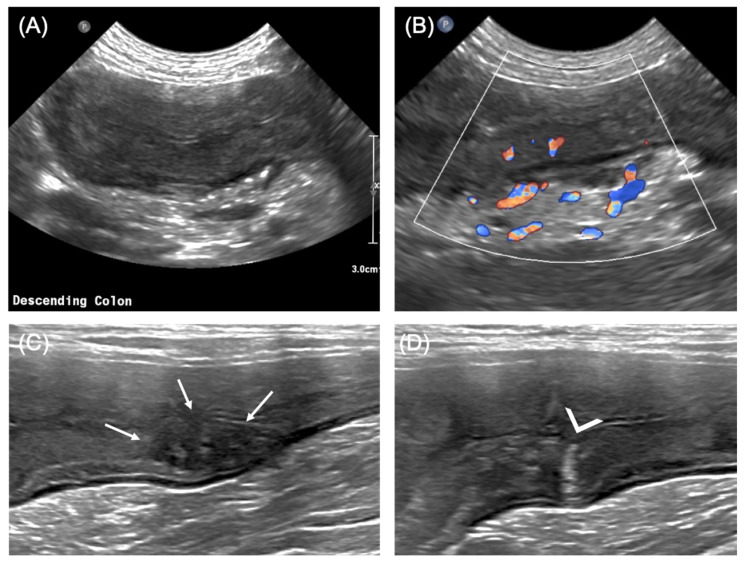
Bi-dimensional US features of the descending colon. (**A**) Appearance of the descending colon, transverse view; note the diffusely thickened wall, with partial loss of layering; (**B**) color Doppler flow at the level of the descending colon showing increased flow both within the wall and in the surrounding mesentery; (**C**) heterogeneously hypoechoic nodule in the colonic wall (arrows), apparently involving the mucosal and submucosal layers; (**D**) reverberating spot (arrowhead) arising from the mucosal surface at the level of the colonic wall, most likely representing a superficial ulceration. (**A**,**B**): micro-convex transducer (5–8 MHz); (**C**,**D**): linear transducer (8–14 MHz).

A US examination of the abdomen was performed with an Epiq 7 (Philips) ultrasound machine equipped with both a micro-convex (frequency 5–8 MHz) and a high-frequency linear transducer (4–18 MHz). The descending colon was diffusely and markedly thickened (maximum thickness of approximately 10 mm), with reduced detail of the normal layering and marked thickening of the mucosa in other portions (Figure 1A). The colonic wall and the surrounding mesentery were characterized by increased vascularity, as demonstrated by the color Doppler evaluation (Figure 1B). Some hypoechoic nodular structures were seen in the wall, and multiple reverberating spots were noticed in the inner layers (Figure 1C,D). The ascending and transverse portions of the colon showed a normal US appearance. The caecum had a diffusely thickened wall (6 mm) with similar characteristics as those described for the descending colon. The left colic lymph nodes were enlarged (6 mm) and heterogeneous.

As differential diagnoses, both severe inflammatory process and infiltrative neoplasia (as lymphoma) were considered.

For a better characterization, contrast-enhanced ultrasonography of the descending colon was performed (4–12 MHz linear transducer). An ultrasound contrast agent (Sonovue, Bracco, Milan, Italy; 0.05 mL/kg body weight) was injected as a bolus in the left cephalic vein, followed by 5 mL of physiologic saline (0.9% NaCl, Mini-Plasco, B.Braun, Oss, the Netherlands).

**Figure 2:**
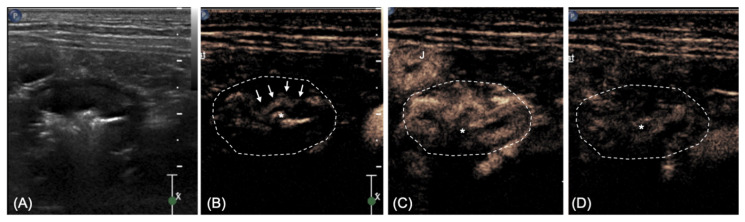
CEUS appearance of the descending colon at three different time points. (**A**) Bidimensional image for comparison; (**B**) arrival of the contrast media at the level of the inner layers (arrows) 9 s after injection; (**C**) peak of enhancement, more prominent at the level of the inner layers (17 s); (**D**) heterogeneous wash-out at 30 s. The margins of the colon are highlighted for more clarity. * lumen of the colon.

The perivisceral vessels showed rapid enhancement, with enhancement of the colon starting from the inner layers (Figure 2A). Maximum enhancement was reached after 17 s (Figure 2B) showing heterogeneous distribution within the wall, with the outer layers (muscularis, serosa) less enhanced compared with the inner layers (mucosa, submucosa). After this peak in enhancement, the wall gradually showed reduced enhancement, with only residual portions enhanced after 30 s (Figure 2C). The pattern of enhancement was therefore defined as a rapid wash-in and gradual wash-out.

**Figure 3:**
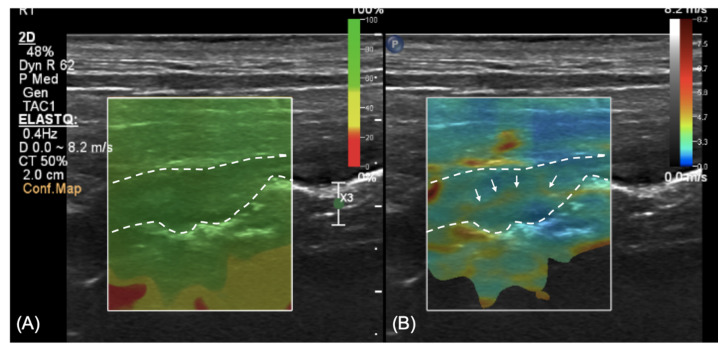
Shear-wave elastography at the level of the descending colon. (**A**) Confidence map to assess the reliability of the measurements; green color represents a reliable zone; (**B**) elastography at the level of the wall showing a predominance of blue areas (soft) with a yellow (harder) thin line running within it (arrows).

A qualitative evaluation using shear-wave elastography with a linear transducer (4–18 MHz) was also performed at the level of the descending colon. A colored confidence map (ElastQ, Philips, Germany) was used to assess the reliability of the shear-wave analyses (Figure 3A). The elastography pattern of the colonic wall was mixed, with predominantly blue areas (soft) and a consistent presence of yellow areas at the level of the outer mucosa, presumably corresponding to the lamina propria (Figure 3B).

Both CEUS and elastography were performed on the conscious patient using manual restraint. No sedation was necessary to achieve good image quality. The CEUS and elastography pattern were subjectively considered primarily consistent with an inflammatory process, although (due to the lack of reference literature for comparison) a neoplastic process could not be ruled out.

### 2.3. Endoscopy, Histopathology, Treatment, and Outcome

**Figure 4:**
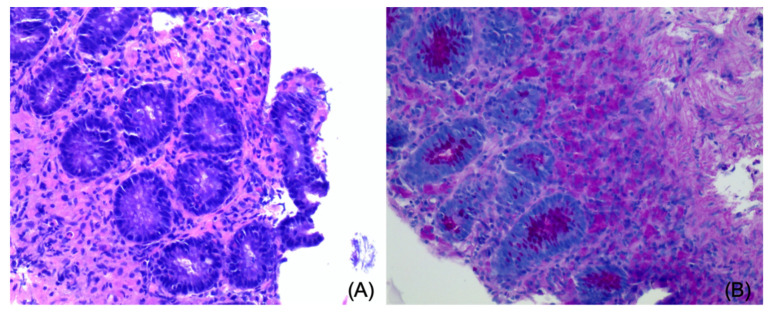
Histology of the colonic wall. (**A**) Moderate amount of edema and fibrosis of the lamina propria (Hematoxylin and Eosin); (**B**) presence of a dense infiltrate of strong PAS-positive macrophages (PAS).

The dog underwent a colonoscopy on the same day under general anesthesia. The colonic mucosa was considered thickened and fragile, with multiple hyperemic areas and some superficial erosions, both at the level of the descending colon and the caecum. Several biopsies were taken for further histological evaluation. Histology revealed the presence of several mucosal ulcerations covered with a thick layer of fibrin and neutrophils; the epithelium was moderately hyperplastic and covered with mucus. Varying degrees of fibrosis were noticed at the level of the lamina propria (Figure 4A), and a preponderance of PAS-positive macrophages was also detected (Figure 4B).

These findings were considered compatible with granulomatous (or ulcerative) colitis. Furthermore, the cytologic evaluation revealed the presence of intramucosal bacteria (cocci); a confirmation of E. coli infection was not obtained.

The dog started medical treatment with enrofloxacin (10 mg/kg SID) for 10 weeks, with complete resolution of the clinical presentation after 2 weeks of treatment. Three months after the diagnosis, the dog is in good clinical condition with no symptoms related to the gastrointestinal tract. A US follow-up was suggested but declined by the owner.

## 3. Discussion

Granulomatous colitis, previously called ulcerative colitis or histiocytic ulcerative colitis, is an inflammatory condition of the large intestine described in young Boxers, French Bulldogs [1,2,4,6,7], and sporadically in some other breeds [3,15].

Clinical presentation is typically large intestine inflammation, and the most common signs are frequent diarrhea, hematochezia, and tenesmus [1,2]. Although clinical and histological findings have been previously described in detail along with the role of invasive Escherichia coli and the subsequent response to antimicrobial treatment [1,2,4,6], imaging findings regarding this specific pathology are lacking. In this report, we describe the two-dimensional, CEUS, and elastography findings in a French Bulldog with confirmed granulomatous colitis. Inflammatory conditions of the colon can be visualized with US as a mild thickening of the submucosal layer, in which hypoechoic nodules, consistent with lymphatic follicles, can be seen [16]. In the case of ulcerative colitis, the wall is described as thickened, and the mucosal surface is irregular due to superficial erosions [2,9].

In the case reported here, the wall of the descending colon was extremely thickened (up to 10 mm), with reduced detail of the normal layering. Where the layering was still visible, the mucosa appeared to be markedly thickened and hyperechoic, as previously described [9]. Some reverberating spots were also present in the inner layers of the wall, confirming the ulcerative component, and increased color Doppler flow was interpreted as a sign of severe inflammation. Additionally, some large heterogeneous hypoechoic structures were seen in the thickness of the colonic wall; to date, this feature has not been described in dogs with granulomatous colitis. These nodules may represent granulomas of the wall, which are typically present in this disease and were visible in our case due to their large dimensions and the use of a high-frequency linear transducer. Another hypothesis is that these nodules represent the lymphatic follicles described at the level of the submucosa [16], reaching conspicuous dimensions because of severe inflammation.

Both in human and veterinary medicine, some US features of severe inflammation and neoplastic infiltration can overlap; in addition, gastrointestinal neoplasia may lead to wall ulceration, making a definitive diagnosis difficult [9]. Because of its proven safety, availability, and lack of radiation, in human medicine, CEUS is widely used in the clinical setting to diagnose and monitor inflammatory bowel diseases, including ulcerative colitis and Crohn’s disease [12,13]. The distribution of the vessels in inflammatory lesions is reported to have a typical “comb-teeth-like” distribution, but in some cases of severe inflammation, this feature is no longer distinguishable [12]. The CEUS enhancement seen in this case reflects the described enhancement of the duodenum in dogs with IBD and lymphoma [17]. The outer layer in our case and the muscularis in the study of Nisa et al. were markedly less enhanced than the mucosa. In the case of granulomatous colitis, the mucosa and submucosa are known to be the more affected layers [1]. Furthermore, a rapid wash-in and gradual wash-out pattern was detected in our patient, similar to that described for the duodenum (in the normal, inflammatory, and neoplastic groups) [17].

A recent study reported normal stiffness of the mucosa in dogs as assessed by real-time shear-wave elastography [18], but a normal elastography pattern of the colon in a dog has not been described to date. A qualitative assessment of the intestinal wall using a color map was described in children with Crohn’s disease; a normal wall has a “coloured stratification” pattern, with the mucosa and muscularis stiffer than the submucosa, whereas in more severe cases, the wall is uniformly “stiff”, reflecting extensive fibrosis of the intestine [14]. In the case reported here, a thin linear area of increased stiffness (colored in yellow in our color mapping setting) was constantly seen in the central portion of the thickened wall, while the remainder of the wall was homogeneously soft (colored in blue). We hypothesize that this linear thin layer of increased stiffness of the tissue can be explained by the presence of fibrosis at the level of the lamina propria, as described in the histological evaluation. Nevertheless, due to the lack of normal reference values regarding elastography in the canine colon and the impossibility to clearly distinguish the lamina propria with two-dimensional US, this finding has to be evaluated cautiously.

This is a single case reporting the use of CEUS and elastography in the characterization of large intestine enteropathy in a dog with granulomatous colitis. A US follow-up after the treatment would have been useful in this case to determine if the clinical resolution of the symptoms corresponded to an improvement or resolution of the US findings. The confirmation of E. coli infection, although supported by the cytological findings and the complete clinical resolution of the symptoms after antimicrobial treatment, was not achieved with fluorescence in situ hybridization (FISH) as suggested by the literature [19]. Further studies in a larger population of dogs are needed to assess the clinical utility of CEUS and elastography in the workup of dogs with granulomatous colitis.

## 4. Conclusions

In conclusion, both CEUS and elastography were non-invasive techniques that were relatively easy to perform with no need for sedation. These promising techniques may be helpful in the characterization of granulomatous colitis in dogs.

## Data Availability

Data sharing is not applicable to this article.
